# Human papillomavirus genotypes distribution in cervical cancer cases in Gabon

**DOI:** 10.1186/s13027-016-0091-8

**Published:** 2016-08-16

**Authors:** Samira Zoa-Assoumou, Angelique Ndjoyi-Mbiguino, Barthelemy Mabika Mabika, Ernest Belembaogo, Abdelkim Khattabi, My Mustapha Ennaji

**Affiliations:** 1 Laboratoire National de Référence IST/Sida, Laboratoire de Référence OMS Rougeole, Rubéole, Fièvre jaune et Tétanos néonatal, Département de Bactériologie-Virologie, Faculté de Médecine et des Sciences de la Santé, Université des Sciences de la Santé, Libreville, Gabon; 2 Laboratoire de Virologie, Microbiologie et Qualité/Eco-toxicologie et Biodiversité, Faculté des Sciences et Techniques de Mohammedia, Université Hassan II-Casablanca, Casablanca, Morocco; 3 Département d’Anatomie et de Cytologie pathologiques, Faculté de Médecine et des Sciences de la Santé, Université des Sciences de la Santé, Libreville, Gabon; 4 Institut de Cancérologie d’Agondjé, Libreville, Gabon; 5 Laboratoire d’Agroalimentaire et Santé, Département de biologie, Faculté des Sciences et Techniques de Settat, Université Hassan I, Settat, Morocco

**Keywords:** Human papillomavirus, Cervical cancer, Genotyping, Gabon

## Abstract

**Background:**

Cervical cancer is a real public health problem in African countries. The relation between HPV and cervical cancer is well established. However, it is known that the distribution of HPV genotypes differ geographically and this may influence the effectiveness of the three available vaccines, which among other HPV genotypes targets the genotypes 16 and 18 that cause about 70 % of cervical cancers cases. The objective of this study was to identify for the first time the HPV genotypes distribution in cervical cancer specimens obtained from Gabonese women.

**Methods:**

A total of 105 cervical samples including 93 formalin-fixed paraffin embedded tissues collected between 2007 and 2013 and 12 fresh biopsies collected in August 2013 were investigated. The presence of HPV DNA was analyzed by nested PCR with primers MY09/11 and GP5+/6+ followed by sequencing for HPV genotyping.

**Results:**

Amplification of the housekeeping gene (β-globin) with PCO4/GH20 primers was successful for 91.4 % (96/105) of the cervical cancer samples and HPV DNA was detected in all the 96 samples. Five different HPV genotypes were identified. HPV 16 [58.3 %; 95 % IC: 48.44–68.16] was the most common genotype followed by HPV 33 [25.0 %; 95 % IC: 16.34–33.66], HPV 18 [8.4 %; 95 % IC: 2.86–13.94], HPV 70 [7.3 %; 95 % IC: 2.1–12.5] and HPV 31 [1.1 %; 95 % IC: −0.986–3.186]. HPV 16 was also the most prevalent in all histological malignant lesions. It was found in 56.6 % of squamous cervical carcinoma and 69.2 % of adenocarcinoma. Concerning the HPV positive adenocarcinoma cases, HPV 18 was identified in 7.7 % (1/13).

**Conclusion:**

These findings show the predominance of HPV 16 in cervical cancer cases among Gabonese women. However, HPV33 is more prevalent than HPV18. Our study suggests that HPV vaccines may be effective at reducing the burden of cervical cancer in Gabon.

## Background

Infection with high risk oncogenic Human Papillomavirus (HPV) plays a necessary role in the development of cervical cancer, which is the third most frequent gynecological malignancy in women worldwide [1]. It has been estimated that about 529,800 new cases and 275,100 deaths occurs each year [1]. The burden of this disease is more pronounced in developing countries where it is the second frequent female cancer after breast cancer [1]. In Gabon, a Sub-Saharan country, cervical cancer is the most frequent cancer in women [2]. According to data from the International Agency for Research on Cancer in 2012 [3], the age-standardized incidence of cervical cancer among Gabonese woman is 19.9 per 100, 000 women and the mortality is 8.4 per 100,000.

In the fifteen high risk HPV genotypes, HPV16 and 18 are the two genotypes found in approximately 70 % of cervical cancer cases worldwide [4]. Thus, the two preventive vaccines available (Cervarix® and Gardasil®) were developed by taking into account the global prevalence of these two oncogenic genotypes. However, many studies on HPV type distribution have shown some geographical variation [5, 6], suggesting that the usefulness of these HPV vaccines, in some region of the world, may be low. Therefore, one of the keys to a successful vaccination based preventive strategy would be the knowledge of the distribution of HPV types, both among cervical cancer cases and in the general population. This information is also needed for a virological surveillance of HPV distribution among the general population before and after the vaccination.

To date, no study, related to HPV genotypes in cervical cancers has been conducted in Gabon. Thus, the aim of the present study was to determine the HPV genotypes distribution in cervical cancer specimens obtained from Gabonese women.

## Methods

### Acquisition of cervical carcinoma specimens

A total of 105 biopsies were investigated. These included 93 Formalin-Fixed Paraffin Embedded (FFPE) tissues listed between January 2007 and August 2013 at the Department of Anatomy and Cytology of the University of Health sciences of Libreville in Gabon and 12 fresh biopsies collected from the Cancer Institute of Libreville in August 2013. The only inclusion criterion was availability of information on the pathological diagnosis. Histology results of samples collected showed 92 Squamous Cervical Carcinoma (SCC) and 13 Adenocarcinoma (ADC).

### DNA extraction and quantification

Four sections of 3 μm were cut on a microtome under strict conditions and deparaffinized with a high-temperature treatment as described by Steinau et al. [7] and for the fresh biopsies, two washes with phosphate buffered saline were done. Then, 200 μL of lysis buffer (1 M Tris-HCl pH 8, 0.2 M EDTA, 10 % SDS, 5 M NaCl) containing 10 mg/ml of proteinase K was added to each tube. The tubes were then incubated at 65 °C overnight. DNA was isolated using the standard phenol chloroform method. Then, the DNA was resuspended in nuclease free water (Bioline, UK) and stored at −20 °C until further use. After extraction, DNA quantification was performed through nanodrop 8000 Spectrophotometer (Thermo Scientific, Wilmington, DE, USA).

### HPV detection and genotyping

To evaluate the efficiency of the extraction, integrity of specimen and absence of PCR inhibitors, all extracted DNA were subject to an amplification of β-globin reference gene using the primers pair PCO4/GH20 [8] (Table 1).

**Table 1:** Primers used for HPV detection and genotyping

Primers	Sequences (5′ to 3′)	Target gene	Amplicons length
PC04	CAA CTT CAT CCA CGT TCA CC	β-globin	268 bp
GH20	GAA GAG CCA AGG ACA GGT AC		
MY09	CGT CCM ARR GGA WAC TGA TC	L1	450 bp
MY11	GCM CAG GGW CAT AAY AAT GG		
GP5+	TTT GTT ACT GTG GTA GAT ACT AC	L1	142 bp
GP6+	GAA AAA TAA ACT GTA AAT CAT ATT C		
	M = A + C, R = A + G, W = A + T, Y = C + T		

HPV detection and typing was carried out by nested PCR using the L1 consensus primers MY09/11 and GP5+/6+ [9] (Table 1) and DNA direct sequencing. The MY and GP+ primers amplified respectively a fragment of 450 and 150 bp. PCR reactions were performed in a total volume of 25 μl of the reaction mixture containing 10 mM of dNTP, 2.5 mM of Mgcl_2_, 0.2 U of Go*Taq* DNA polymerase, and 10 μM of MY or GP+ primers in 1X *Taq* polymerase buffer. For the GP+ PCR, 2 μl of the MY PCR products was used as template. PCR amplification was performed in a Perkin Elmer 2400 GeneAmpR® PCR thermal Cycler (Scientific Support, Inc, Hayward, CA), and was started with an initial denaturation step (95 °C for 10 min), followed by 40 cycles of 95 °C for 1 min, annealing temperature (55 °C for MY primers and 48 °C for GP+ primers) for 1 min and 72 °C for 1 min; a final extension of 7 min at 72 °C was performed. For every reaction, ultrapure water nuclease free (Bioline, UK) was used as a negative control and DNA of SiHa cell lines was used as positive control. Amplified PCR products were analyzed on a 2 % agarose gel stained with Ethidium bromide and visualized by UV light.

HPV genotyping was performed by DNA sequencing. The PCR products were purified using the ExoSaP-IT clean up system (USB, USA) and the sequencing reaction was performed using GP6+ primer as the sequencing primer with the BigDye Terminator v3.1 Cycle Sequencing kit (Applied Biosystems, Foster City, CA, USA) on an ABI 3130 XL DNA analyzer (Applied Biosystems, Foster City, CA, USA) according to manufacturer’s protocol.

The sequences were analyzed by MEGA software version 6.0.5 (www.megasoftware.net) and results were analyzed using the BLAST server (http://www.ncbi.nlm.nih.gov/blast/) available in GenBank database (NCBI, National Institute of Health, Bethesda, MD, USA). A hypervariable region from 34 to 50 bp downstream of the GP5+ binding site was required for identified any HPV genotype [9]. At least 90 % identities matching between the query and subject sequences were required for genotyping [9].

## Results

### HPV DNA prevalence

Amplification of the housekeeping gene (β-globin) with PCO4/GH20 primers was successful for 91.4 % (96/105) cervical cancer samples (9 FFPE and 1 fresh biopsy) and therefore were eligible for the further analyses. HPV DNA was detected in all the 96 cervical cancer samples positive for β-globin detection. According to the histopathological distribution, HPV detection showed that 90.2 % (83/92) of SCC cases and the entire ADC cases were positive for HPV DNA (Table 2).

**Table 2:** Distribution of HPV genotypes by histopathological categories

		Type of malignant lesions				
	All samples (*n* = 96)	95 % IC	ADC (*n* = 13)	95 % IC	SCC (*n* = 83)	95 % IC
HR^a^ HPV+	89 (92.7)		13 (100)		76 (91.6)	
Pc^b^ HPV+	7 (7.3)		0 (0.0)		7 (8.4)	
HR genotypes						
HPV 16	56 (58.3)	48.4–68.2	9 (69.2)	44.1–94.3	47 (56.6)	45.9–67.3
HPV 33	24 (25.0)	16.3–33.7	3 (23.1)	0.2–46	21 (25.3)	15.9–34.7
HPV 18	8 (8.3)	2. 9–13.9	1 (7.7)	−6.8–22.2	7 (8.4)	2.4–14.4
HPV 31	1 (1.1)	−0.9–3.1	0 (0.0)	–	1 (1.2)	−1.1–3.5
Pc^b^ genotypes						
HPV 70	7 (7.3)	2.1–12.5	0 (0.0)	–	7 (8.4)	2.4–14.4

Percentage is in bracket. ^a^High Risk. ^b^Possibly carcinogenetic

### HPV genotype prevalence and distribution

Analysis of sequencing results showed that 4 oncogenic HPV genotypes: 16, 18, 33 and 31, and one possibly carcinogenetic genotype, HPV 70 were present in the cervical cancer specimens studied. HPV 16 was the most prevalent genotype representing 58.3 % [95 % IC: 48.44–68.16] of HPV positive cases followed by HPV 33 [25.0 %; 95 % IC: 16.34–33.66], HPV 18 [8.4 %; 95 % IC: 2.86–13.94], HPV 70 [7.3 %; 95 % IC: 2.1–12.5] and HPV 31 [1.1 %; 95 % IC: −0.986–3.186]. In SCC and ADC cases, the prevalence of HPV 16 was 56.6 % (47/83) and 69.2 % (9/13) respectively. HPV 18 was detected in 7.7 % (1/13) of ADC and in 8.4 % (7/83) of SCC cases. All the HPV 70 cases found in this study were SCC (Table 1).

## Discussion

In the WHO middle African countries, data about HPV distribution in relation to cervical abnormalities are still lacking. This work is the first comprehensive study conducted in Gabon that investigated the distribution of HPV genotypes in invasive cervical cancer.

In Gabon, as reported in a study from Congo-Brazzaville [10], a neighbouring country, the number of pathological laboratories are really limited. Furthermore, the specimens used in this study were obtained from women who came from different regions of the country and represents a wide social and economic strata. Therefore, the results of this study could reflect the trend distribution of oncogenic HPV among cervical cancer cases in our country.

Worldwide HPV prevalence in cervical carcinomas is reported to be 99.7 % and cases of cervical cancer without HPV is extremely rare [11]. Of the 96 cervical cancer specimens genotyped, all were determined as positive for HPV DNA. This high HPV prevalence found corroborate with an international study conducted by Sanjose S et al. [12] that used FFPE tissues collected from the five continents with slight differences depending on the region. Some others African studies reported a high HPV prevalence. In a Sudanese and Ethiopian study, [13] the HPV prevalence was respectively 94 and 93.1 % of cervical carcinoma cases. In a sub-Saharan study [14] conducted with cervical samples from Ghana, South Africa and Nigeria, the HPV prevalence was 93.9; 92.1; and 84.9 % respectively. Another study conducted in Ghana [15] reported a high prevalence of 98 % in cervical cancer cases.

Correlation between histological status and HPV genotypes has been observed in several studies. SCC was most likely associated with HPV 16 whereas HPV 18 was most common in ADC [16–18]. However, in our study, HPV 16 was the most prevalent in all histological categories of cervical cancer cases. Indeed, this HPV genotype was found in 69.2 % of ADC and in 58.3 % of SCC. However, HPV 18 was found in only one case (7.7 %) of ADC and in 8 cases (8.3 %) of SCC. Our results suggest that HPV 16 remains prevalent whatever the histological type in our population of cervical cancer cases and highlight the oncogenic potential of this genotype.

In our study, we found 4 of these oncogenic genotypes. HPV 16 was the most predominant type in all HPV positives samples representing 58.3 % of cases followed by HPV 33 (25 %). The findings of this study confirms the fact that there is a geographical variations in the genotype specific HPV distribution globally [19]. The distribution determined in this study was similar to those reported in some African studies [10, 20, 21], while for others African countries the distribution was different [5, 13, 14].

In a recent upgraded IARC systemic review about human carcinogens [22], HPV 70 earlier considered as a low-risk HPV genotype has been considered as a possibly carcinogenetic genotype. In our study, this HPV genotype was found in 7.4 % of the cervical cancer samples. This genotypes was found in cervical cancer cases in several studies [14, 23–26]. Our findings highlight the fact that an epidemiological surveillance would be necessary for this uncommonly genotype in our population.

The HPV genotypes distribution found in this study differ from those of the preliminary study among Gabonese women of childbearing age, regardless of their cervical cytology [27]. In fact, the oncogenic genotypes 53, 58 and 16 were found to be the most prevalent in decreasing order. Moreover, HPV 70 was also found in 6 % of HPV positives cases. The absence of the oncogenic genotype 53 and 58 in our study suggest that they may be less commonly associated with cervical cancer in our population.

This study also has a limitation. These specimens could very probably harbor multiple infections and therefore could not be typed by DNA sequencing. DNA sequencing technique has been facing limitation when the specimen harbors multiple genotypes. Other genotyping approach would be used to determine the multiple HPV types in our future studies.

At last, from a prevention point of view, our study suggests that the introduction of HPV vaccination may reduce the burden of cervical cancer in Gabon and economic efforts in the treatment of this preventable disease.

## Conclusion

This study provides the first information about the HPV distribution in cervical cancer cases in Gabonese women. Our study showed that HPV 16, 33 and 18 were the most common genotypes in women with SCC and ADC. The determination of HPV distribution is important for implementing programs of vaccination and virological surveillance. Our findings highlight that the introduction of HPV vaccination in Gabon should be considered and will offer a significant opportunity for the Gabonese National Health Program to control cervical cancer disease and save women lives.

## Abbreviations

ADC, adenocarcinoma; HPV, human papillomavirus; SCC, squamous cervical carcinoma
